# Optimizing composite PET measurements for tracking longitudinal tau accumulation

**DOI:** 10.1002/alz.090463

**Published:** 2025-01-09

**Authors:** Hillary D. Protas, Valentina Ghisays, Ji Luo, Javad Sohankar, Wendy Lee, Vivek Devadas, Kewei Chen, Eric M. Reiman, Yi Su

**Affiliations:** ^1^ Banner Alzheimer's Institute, Phoenix, AZ USA; ^2^ Arizona Alzheimer's Consortium, Phoenix, AZ USA; ^3^ School of Mathematics and Statistical Sciences, College of Health Solutions, Arizona State University, Tempe, AZ USA; ^4^ Department of Neurology College of Medicine‐Phoenix, University of Arizona, Phoenix, AZ USA; ^5^ Biodesign Institute, Arizona State University, Tempe, AZ USA; ^6^ Department of Psychiatry, University of Arizona, Phoenix, AZ USA; ^7^ University of Arizona College of Medicine Phoenix, Phoenix, AZ USA; ^8^ ASU‐Mayo Center for Innovative Imaging, Tempe, AZ USA

## Abstract

**Background:**

Tangle burden, one of the hallmarks of Alzheimer’s Disease, is thought to accumulate and spread throughout the brain in a distinctive pattern starting from the entorhinal cortex following Braak stages as characterized in neuropathological studies. Longitudinal tau PET allows us to investigate in vivo the tau spread in an individual and substantial heterogeneity has been observed in the pattern of tau spread. In this analysis, we examine the statistical power of tau PET measurements in tracking disease progression using data from the ADNI cohort.

**Method:**

The tau PET measures used in this study included conventional SUVR measures as well as graph theory based network strength measures as we previously reported (Protas 2023). We calculate the slopes of both regional SUVR and strength in 41 MCI and 71 CU participants who had one year, and/or two year follow‐up. Statistical power was assessed as the sample size needed to detect a 25% reduction in the rate of tau accumulation, and the power analysis was done for CU and MCI participants separately. The optimal composite measures (from combining up to 6 FreeSurfer ROIs) were determined based on two criteria: 1) having minimal tau accumulation (calculated sample size > 10000) in the A‐ group to ensure we are looking at AD related accumulation, and 2) having the smallest sample size in the A+ group.

**Result:**

Strength in amygdala, entorhinal, fusiform, rostral anterior cingulate, frontal pole and transverse temporal regions gives optimal sample sizes of 172 in A+ CU. In CU A+, SUVR in amygdala, lateral occipital and middle temporal produced an optimal sample size of 230. In MCI, for strength, the sample size is 193(A+) in an optimized region including cuneus, inferior temporal, postcentral, rostral anterior cingulate, and superior parietal. With SUVR the optimal region in MCI was entorhinal and middle temporal with a sample size of 263(A+).

**Conclusion:**

The optimal composite regional SUVR/strength measure varies depending on the target population and typically combines both early and late Braak stages. Further study is needed to confirm this finding using larger datasets.

